# *Helicobacter pylori* Seroprevalence and Its Associations with Sociodemographic Characteristics, Environmental Factors, and Gastrointestinal Complaints: A Cross-Sectional Study in the Adult Population of Kaunas City, Lithuania

**DOI:** 10.3390/medicina61061049

**Published:** 2025-06-06

**Authors:** Paulius Jonaitis, Janina Petkeviciene, Violeta Salteniene, Egle Ciupkeviciene, Laimas Jonaitis, Mantas Kriukas, Dalia Luksiene, Vaiva Lesauskaite, Juozas Kupcinskas, Limas Kupcinskas

**Affiliations:** 1 Department of Gastroenterology, Lithuanian University of Health Sciences, Eiveniu Str. 2, 50009 Kaunas, Lithuania; laimas.jonaitis@lsmu.lt (L.J.); mantas.kriukas@lsmu.lt (M.K.); juozas.kupcinskas@lsmu.lt (J.K.); limas.kupcinskas@lsmu.lt (L.K.); 2 Health Research Institute, Lithuanian University of Health Sciences, Tilzes Str. 18, 47181 Kaunas, Lithuania; janina.petkeviciene@lsmu.lt (J.P.); egle.ciupkeviciene@lsmu.lt (E.C.); 3 Institute for Digestive Research, Lithuanian University of Health Sciences, Eiveniu Str. 4, 50103 Kaunas, Lithuania; violeta.salteniene@lsmu.lt; 4 Institute of Cardiology, Lithuanian University of Health Sciences, Sukileliu av. 15, 50103, Kaunas, Lithuania; dalia.luksiene@lsmu.lt (D.L.); vaiva.lesauskaite@lsmu.lt (V.L.)

**Keywords:** *Helicobacter pylori*, seroprevalence, Lithuania, IgG antibodies, Europe, sociodemographic factors, dyspeptic symptoms

## Abstract

*Background and Objectives*: *Helicobacter pylori*, classified as a Group I carcinogen, is the main risk factor for gastric cancer, one of the leading causes of cancer mortality globally. Lithuania reports one of the highest gastric cancer rates in Europe, yet recent large-scale epidemiological data on *H. pylori* prevalence are lacking. This study aimed to assess the current seroprevalence of *H. pylori* in Lithuanian adults and its associations with sociodemographic, environmental factors, and dyspeptic symptoms. *Materials and Methods*: A cross-sectional study was conducted between 2020 and 2023 at the Lithuanian University of Health Sciences in Kaunas city. Randomly selected adults aged 25–69 years underwent venous blood sampling for *H. pylori* IgG antibody testing (Serion ELISA) and completed a questionnaire on demographic–environmental factors and dyspeptic symptoms in the past 30 days. Subjects previously treated for *H. pylori* were excluded from seroprevalence analysis. Seroprevalence was compared across age groups using χ^2^ and Z-tests with Bonferroni correction. Multivariable logistic regression identified factors associated with *H. pylori* seropositivity. The selected level of statistical significance was *p* < 0.05. *Results*: A total of 1046 adults (mean age 47.2 years, SD = 11.5; 50% males) participated in the study. The overall age-standardized *H. pylori* seroprevalence was 63.1% (95% CI 60.4–66.7). Seropositivity increased with age, peaking at 80.3% in males aged 55–69. Higher seroprevalence was observed among those with basic education and those lacking access to municipal or heated water during childhood. Regression analysis revealed that male sex, aging, and lower education were significantly associated with *H. pylori* seropositivity. No significant link was found between *H. pylori* seroprevalence and gastrointestinal complaints. *Conclusions*: *H. pylori* seroprevalence remains high in Lithuanian adults, highlighting the need for ongoing surveillance and consideration of screening strategies. *H. pylori* infection was linked to sociodemographic and environmental factors but not dyspeptic complaints.

## 1. Introduction

*Helicobacter pylori* (*H. pylori*) is a Gram-negative bacterium that colonizes the human stomach and has the ability to persist for decades. It is recognized as one of the most common chronic bacterial infections worldwide. Even though many carriers remain asymptomatic, chronic infection may lead to severe gastric diseases. This bacterium is the primary cause of chronic gastritis and is associated with numerous conditions, such as gastric cancer (GC), peptic ulcer disease (PUD), mucosa-associated lymphoid tissue lymphoma, and others [1,2,3]. Moreover, *H. pylori*, which was classified as a definite (Group I) carcinogen by the International Agency for Research on Cancer (IARC) in 1994, is the biggest risk factor in the etiopathogenesis of non-cardia GC—one of the leading causes of cancer-related deaths globally [4]. According to the Global Cancer Observatory (GLOBOCAN) database from 2022, GC ranks fifth in both incidence and mortality among various types of cancer [5].

Although the prevalence of *H. pylori* globally is declining, it is estimated that approximately 40–50% of the World’s population is still infected with this bacterium. Higher prevalence rates are observed in developing countries with poor sanitation and crowded living conditions, as well as low socioeconomic status, whereas developed Western countries have lower prevalence rates [3,6,7]. This bacterial infection is typically acquired in childhood through an oral–oral or fecal–oral transmission route. Socioeconomic factors such as sanitation, access to clean water, and education level influence the risk of acquiring *H. pylori* infection, while lifestyle factors like diet, smoking, and alcohol consumption may affect the progression of *H. pylori*-associated conditions [8].

There is a lack of epidemiological data on *H. pylori* in Eastern and Central European countries, including Lithuania. A population-based epidemiological study in Latvia revealed a high (79.2%) prevalence of *H. pylori* infection [9]. In the Tartu population of Estonia, the prevalence of *H. pylori* antibodies was found to be 69% [10]. Similarly, in the neighboring Poland, 78.5% of randomly selected residents in Lublin tested positive for *H. pylori* [11]. The prevalence of *H. pylori* infection in Lithuania and its associated conditions has been examined in several smaller studies, with rates ranging from 36% in children to approximately 70% in middle-aged adults with dyspeptic symptoms. One of the more recent studies revealed a dramatic decrease in the seroprevalence of *H. pylori* over 25 years among Lithuanian medical students, from 52% in 1995 to 14% in 2020 [12,13,14,15]. However, epidemiological data on the general population in Lithuania remain very limited.

Furthermore, the incidence of GC in Lithuania remains one of the highest in Europe, with an age-standardized rate per 100,000 inhabitants of 18.4 in males and 7.2 in females based on the GLOBOCAN 2022 data [5]. The high burden of GC in Lithuania and neighboring countries highlights the critical public health importance of addressing *H. pylori* infection and the need for ongoing surveillance and effective management strategies [16].

The European guidelines for *H. pylori* diagnostics and treatment are presented in the recently updated Maastricht VI/Florence Consensus Report, which is also followed in Lithuania. These guidelines state that validated serological tests for IgG anti-*H. pylori* antibodies can be used for primary diagnosis in cases when endoscopy is not indicated, and there is no history of previous *H. pylori* eradication [1]. A recent study using data from the European Registry on *H. pylori* Management (Hp-EuReg) in Lithuania concluded that the diagnostics and treatment of *H. pylori* infection only partially met international guidelines [17].

Therefore, this study aimed to assess the seroprevalence of *Helicobacter pylori* infection in the adult population of Kaunas city, Lithuania, and analyze its associations with sociodemographic and environmental factors, as well as dyspeptic symptoms, in a broader cohort.

## 2. Materials and Methods

### 2.1. Study Setting and Ethics

The seroprevalence of *H. pylori* IgG antibodies was assessed in a study entitled “Chronic Diseases and their Risk Factors in the Adult Population”. This study was conducted among residents of Kaunas, the second-largest city in Lithuania, aged 25 to 69 years. A random sample of Kaunas males and females aged 25–69 years, stratified by sex and age, was randomly selected from the Lithuanian population register (*n* = 6000). The study began in 2020 but was interrupted due to the COVID-19 pandemic and resumed in 2023. In total, 3426 individuals participated in the study until 21 June 2024. The response rate was 57.1%. *H. pylori* IgG antibodies were assessed in a subsample of individuals who were screened by 23 June 2023. Invitations were mailed to the selected individuals to come for a health check-up at the Hospital of Lithuanian University of Health Sciences (LUHS) Kaunas Clinics.

The study was approved by the Kaunas Regional Biomedical Research Ethics Committee (protocol number BE-2-49, issued on 5 June 2018). Written informed consent was obtained from all participants.

### 2.2. Study Subjects

By 23 June 2023, blood serum samples from 1046 participants were tested for *H. pylori* IgG antibodies. The *H. pylori* study population comprised 526 males (50.3%) and 520 females (49.7%). The mean age of the participants was 47.2 years, with a standard deviation of 11.5 years.

### 2.3. Data Collection and Variables

All study participants completed a questionnaire that included questions about factors potentially associated with the prevalence of *H. pylori* infection. The questionnaire covered the following factors: (1) social and demographic characteristics, such as sex, age, marital status, level of education, and place of residence during childhood; (2) environmental factors, including the number of family members during childhood, the source of drinking water, and access to hot tap water in childhood; and (3) dyspeptic complaints. Participants were categorized into four age groups: 25–34, 35–44, 45–54, and 55–69 years old. They were also grouped into three groups based on education level: (1) basic education: primary, incomplete secondary, and secondary education; (2) intermediate education: technical school or vocational training; (3) advanced education: college or university degree (bachelor’s, master’s or doctoral degree).

Participants were asked if they had previously been tested for *H. pylori*. The possible responses were “no”, “yes, the bacterium was not detected”, “yes, the bacterium was detected”, and “I don’t know”. The subsequent question was “If you have been diagnosed with *H. pylori*, have you used any medications to eradicate the bacteria?” The possible answers were “no”, “yes”, and “I don’t know”.

Additionally, participants were asked binary (yes/no) questions if they had experienced dyspeptic complaints within the past 30 days, including epigastric pain, heartburn, nausea/vomiting, diarrhea, and constipation. The intensity of the symptoms was not evaluated.

### 2.4. H. pylori IgG Antibodies Testing

A blood sample was collected from a peripheral vein to test for *H. pylori* IgG antibodies. Human IgG antibodies against *H. pylori* were detected in serum using a diagnostic quantitative enzyme-linked immunosorbent assay (ELISA) kit (SERION ELISA^®^ Classic *Helicobacter pylori* IgG, Wurzburg, Germany), following the manufacturer’s protocol. The optical density of the reaction solution was measured using a Sunrise microplate reader (Tecan Trading AG, Zurich, Switzerland) at a wavelength of 405 nm, with a reference wavelength of 620 nm. Measurements were analyzed using Magellan™ Standard software (Tecan Trading AG, Zurich, Switzerland).

### 2.5. Exclusion from Further Analysis

In total, 132 participants answered that they had previously received *H. pylori* eradication therapy. In this study, we had no access to the medical history or records of study participants; therefore, we could not ensure that they had really been previously diagnosed with *H. pylori* infection, received eradication therapy, or if the eradication success had been confirmed. To avoid any inaccurate data, these subjects were excluded from the analysis of current seroprevalence and its associations with other independent factors.

### 2.6. Statistical Analysis

Statistical analysis was performed using the statistical package IBM SPSS Statistics 27.0 (IBM Corp.: Armonk, NY, USA, released 2020).

The prevalence of *H. pylori* IgG antibodies was expressed in percentages. The proportions of *H. pylori* seropositive individuals in different groups were compared using a χ^2^ test and Z-test with Bonferroni correction for multiple comparisons. To calculate the total prevalence of *H. pylori* IgG antibodies, the indicators were standardized by age using the age structure of the Kaunas city population as the reference.

A logistic regression analysis was conducted to assess the association between *H. pylori* antibody positivity (the dependent variable) and various sociodemographic and environmental factors. Initially, separate models were created for each variable. Following that, all variables—such as sex, age, education, and childhood environmental conditions (including living location, access to tap water, and hot water supply)—were combined into a single model for multivariable analysis. The selected level of statistical significance was *p* < 0.05.

## 3. Results

### 3.1. Demographic and Socioeconomic Data

A total of 1046 residents from Kaunas participated in the *H. pylori* seroprevalence study. The largest age group was 55–69 years old (30.3%), comprising 28.9% of total males and 31.7% of total females. The smallest age group was 25–34 years old (17.1%), accounting for 19.2% of total males and 15% of total females. Over half (59%) of the participants had attained advanced education. Notably, 75.8% of the participants reported having grown up in urban areas and having access to centralized drinking water and hot tap water during childhood. Additionally, almost half of the study participants (47.9%) grew up in households with four members. Detailed demographic and environmental data are shown in Table 1.

### 3.2. Seroprevalence of H. pylori IgG Antibodies

The age-standardized seroprevalence of *H. pylori* IgG antibodies in the population of Kaunas city, aged 25–69 years, was 63.1% (95% CI 60.4–66.7). *H. pylori* seroprevalence was higher in males (66.3%) compared to females (60.2%). Statistically significant differences in seroprevalence between males and females were observed in the oldest age group (55–69 years). Overall, the proportion of seropositive individuals increased with age, particularly among males. The highest seroprevalence of IgG *H. pylori* antibodies was observed among men aged 55–69 years (80.3%) and women aged 45–54 years (67.2%). Conversely, the lowest prevalence was found in the youngest age group (25–34 years old), with about 48% in both genders. The detailed seroprevalence of *H. pylori* and the comparison between different age groups is presented in Table 2.

### 3.3. Seroprevalence of H. pylori Antibodies in Relation to the Analyzed Independent Factors

The prevalence of *Helicobacter pylori* IgG antibodies was statistically significantly higher among participants with basic education compared to those with intermediate or advanced education. Participants who had access to tap water and hot water during childhood had a statistically significantly lower prevalence of *H. pylori* compared to those having no access to a municipal water supply or heated water. The place of residence and the number of household members during childhood did not influence the prevalence of *H. pylori* infection. Detailed information is presented in Table 3.

Univariable logistic regression analysis indicated that males had higher odds of *H. pylori* infection compared to females, and the likelihood of infection increased with age (Table 4). The highest odds of infection were found among respondents with the lowest level of education. Additionally, individuals who experienced poor hygienic conditions during childhood, specifically those without access to tap water or a hot water supply, had greater odds of seropositivity.

A multivariable logistic regression analysis confirmed that being male and older age were positively associated with the odds of *H. pylori* seropositivity. In contrast, the odds of infection decreased as education levels increased. Furthermore, the associations between *H. pylori* antibody positivity and the source of drinking water, as well as access to hot water during childhood, were found to be statistically non-significant. Detailed data are presented in Table 4.

### 3.4. History of Previous H. pylori Antibodies Testing and Eradication

Most participants (70.5%) reported that they had never been serologically tested for *H. pylori*. Nearly one in ten (11.6%) had been tested previously but received a negative result, while 15% tested positive. Among those who had been tested, the seroprevalence of *H. pylori* was 56.5% (157 out of 278 participants). Detailed data are presented in Table 5.

### 3.5. Dyspeptic Symptoms and Their Association with H. pylori Seroprevalence

**Figure 1:**
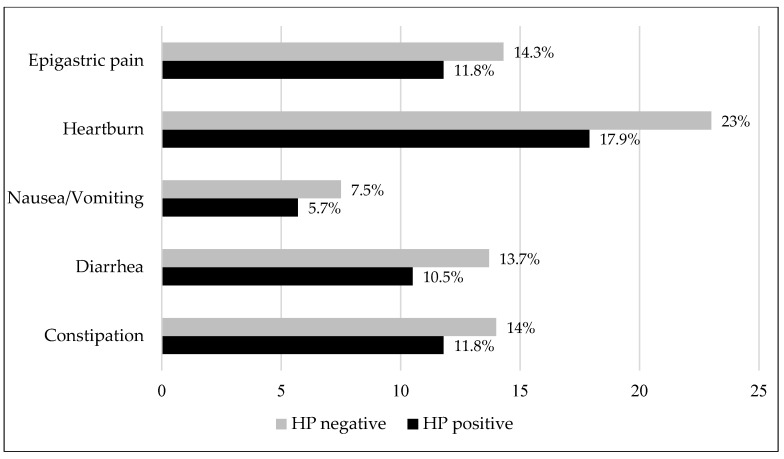
The frequency of gastrointestinal complaints in the past 30 days according to the presence of *H. pylori* antibodies, *p* > 0.05.

Some of the participants reported experiencing gastrointestinal complaints in the past 30 days—around one-fifth of the participants experienced heartburn, which was the most frequent complaint. No statistically significant associations were found between the seroprevalence of *H. pylori* and gastrointestinal complaints, although there was a trend for these complaints to be more prevalent in *H. pylori*-seronegative participants (Figure 1).

## 4. Discussion

The findings of our study reveal the high seroprevalence of *H. pylori* infection in the city of Kaunas, Lithuania, and its associations with sociodemographic and environmental factors, as well as dyspeptic complaints. The overall age-standardized seroprevalence of *H. pylori* in our study (63.1%) likely reflects trends observed in neighboring countries as well; the most recent studies have shown a high *H. pylori* seroprevalence of 55% in Latvia, 84% in Polish adults, and approximately 60% in Ukrainian adults [18,19,20]. Our results confirm that the seroprevalence of *H. pylori* in Lithuania is markedly higher than that seen in Western Europe, where it is ~40%, emphasizing persistent disparities despite socioeconomic improvements. However, based on the previous local studies and the global trend of declining prevalence of *H. pylori*, we can estimate that the actual prevalence of *H. pylori* in Lithuania may be slightly lower than our current study findings suggest [13,14,15]. Some participants, who reported being treatment-naïve, might have actually been prescribed eradication therapy in the past, and a small number of patients were unaware of whether they had been tested for *H. pylori* or prescribed the treatment. A second diagnostic test in the *H. pylori* IgG-positive group could be considered to confirm the diagnosis.

Age-specific analysis in our study confirmed the “cohort effect”—the prevalence was higher among older cohorts. Our findings have shown a steady increase in prevalence with age, peaking at 80.3% in males aged 55–69, with the lowest prevalence in the youngest cohort of 25–34 years old. A higher prevalence in older populations and a declining prevalence among younger people in Lithuania have already been observed in previously published studies [12,13,14,15,17]. This trend reflects differences in exposure to poor sanitation during childhood, aligning with the “cohort effect” observed in global studies, where older populations exhibit higher *H. pylori* infection rates due to poorer socioeconomic conditions in their childhood [21].

Educational attainment emerged as one of the main determinants of *H. pylori* prevalence, with the highest rates observed among participants with basic education compared to those with advanced education. This association is consistent with most studies highlighting education as a proxy for better hygiene, nutrition, and healthcare access [22,23]. Access to drinking water was also associated with *H. pylori* prevalence: participants who relied on drinking water from wells had significantly higher infection rates compared to those using municipal water supplies. Similar trends were observed when evaluating access to hot water; its absence in childhood was associated with significantly higher prevalence. These findings also align with available evidence linking poor water quality and sanitation to higher infection rates. Interestingly, no significant differences were found when evaluating childhood living environment (urban vs. rural), contrary to available global data suggesting higher rates in rural areas [24,25,26,27].

The relationship between *H. pylori* and dyspeptic symptoms has been widely evaluated and discussed in existing studies, and the results are controversial. Even though some studies report a clear relationship between the prevalence of *H. pylori* and the intensity of dyspeptic symptoms [28], other studies do not support these findings [29], which was also a case in our current study.

The high seroprevalence of *H. pylori* in Lithuania, particularly among older and less educated populations, underscores the need for targeted public health interventions to reduce the incidence of GC and its associated health burden. Population screening and treatment for *H. pylori* infection to control gastric cancer has been proposed as a cost-effective strategy in high-risk populations [30,31]. This approach has already been successfully implemented in a few Asian countries [32]. As Lithuania belongs to a European area of high GC incidence, *H. pylori* screening and eradication programs focused on high-risk groups, along with efforts to improve socioeconomic conditions, could significantly reduce infection rates and the burden of GC. Currently, several European Union projects are ongoing in multiple European countries, evaluating the need for GC screening, possible implementation strategies, and providing essential missing evidence [16]. Lithuania is also a consortium member in some of these projects.

Finally, the strengths and limitations of our study need to be noted. Our research contributes to the epidemiological data of *H. pylori* in Lithuania and the surrounding region and is the largest-scale study evaluating the seroprevalence of this bacterial infection in our country to date. The high seroprevalence clearly indicates that a large proportion of the Lithuanian population is at a higher risk of developing GC; therefore, the results of this study might contribute to the process of implementing GC screening strategies in the near future, such as the “screen-and-treat” strategy. On the other hand, one of the main limitations of our research is the lack of access to official medical documentation on *H. pylori* diagnostics and eradication in previously treated subjects. Since we had to take the word of the study participants about any possible previous eradication prescriptions, these subjects were excluded from further analysis. Even though the number of such subjects was low (13%), if we had medical records of previous *H. pylori* diagnostics and treatment, this could have contributed to the evaluation of *H. pylori* IgG antibody titers after the treatment. Another limitation is related to the constraints of serology testing for *H. pylori* infection, as it may indicate a past infection rather than a current positive status for *H. pylori*. While serological testing is suitable for epidemiological studies at the population level, future research using stool antigen testing could provide a clearer understanding of the prevalence of *H. pylori* infection in the country.

## 5. Conclusions

The IgG seroprevalence of *H. pylori* in adult Kaunas residents aged 25–69 years was high. Higher infection rates were detected in respondents with lower educational attainment, lack of access to municipal water supplies, or lack of access to hot water in childhood. Our study confirmed the “cohort effect”: higher seroprevalence was observed in the older population. No association was found between *H. pylori* seroprevalence and dyspeptic symptoms.

The high seroprevalence of anti-*H. pylori* IgG in Lithuania, a country with a high incidence of gastric cancer, further raises the question of whether population-based or age-group-specific GC screening strategies—such as “screen-and-treat” for *H. pylori*—should be introduced to reduce the incidence and burden of GC.

## Figures and Tables

**Table 1:** Sociodemographic and environmental characteristics of the study participants.

Characteristics	Males		Females		Total		*p*-Value Between Males and Females
	*n*	%	*n*	%	*n*	%	
**Age group (years)**							
25–34	101	19.2	78	15.0	179	17.1	0.062
35–44	149	28.3	128	24.6	277	26.5	
45–54	124	23.6	149	28.7	273	26.1	
55–69	152	28.9	165	31.7	317	30.3	
**Level of education**							
Basic	194	37.0	130	25.1	334	31.1	<0.001
Intermediate	40	7.6	63	12.2	103	9.9	
Advanced	291	55.4	325	62.7	616	59.0	
**Place of residence in childhood**							
Urban	405	77.1	386	74.5	791	75.8	0.322
Rural	120	22.9	132	25.5	252	24.2	
**Drinking water from municipal supply in childhood**							
Yes	395	75.2	378	73.0	773	74.1	0.404
No	130	24.8	140	27.0	270	25.9	
**Hot tap water in childhood**							
Yes	398	75.8	370	71.4	768	73.6	0.108
No	127	24.2	148	28.6	275	26.4	
**Number of household members in childhood**							
2–3	112	21.3	101	19.5	213	20.4	0.044
4	266	50.6	234	45.2	500	47.9	
5 and more	148	28.1	183	35.3	331	31.7	

**Table 2:** Seroprevalence of *H. pylori* among different age groups and genders of Kaunas city residents.

Age Groups	Males (*n* = 472)				Females (*n* = 442)				Total (*n* = 914)			
	HP-Negative		HP-Positive		HP-Negative		HP-Positive		HP-Negative		HP-Positive	
	*n*	%	*n*	%	*n*	%	*n*	%	*n*	%	*n*	%
25–34	48	52.2 ^a^	44	47.8 ^a^	37	52.1 ^c^	34	47.9 ^c^	85	52.1 ^c^	78	47.9 ^c^
35–44	50	37.9 ^b^	82	62.1 ^b^	39	35.1	72	64.9	89	36.6	154	63.4
45–54	26	23.4 ^b^	85	76.6 ^b^	39	32.8	80	67.2	65	28.3	165	71.7
55–69	27	19.7 *	110	80.3 *	56	39.7	85	60.3	83	29.9	195	70.1
**Total non-standardized**	151	32.0 *	321	68.0 *	171	38.7	271	61.3	322	35.2	592	64.8
**Total age-standardized, %** **(95% CI)**	33.7 (27.8–36.2)		66.3 (62.3–70.8)		39.8 (34.2–43.2)		60.2 (55.8–65.0)		36.9 (32.1–38.3)		63.1 (60.4–66.7)	

HP: *Helicobacter pylori*; * *p* < 0.05 compared to females; ^a^ *p* < 0.05 compared to the 45–54 and 55–64 age groups; ^b^ *p* < 0.05 compared to the 55–69 age group; ^c^ *p* < 0.05 compared to other age groups (Z-test with Bonferroni correction); 95% CI: 95% confidence interval.

**Table 3:** Prevalence of *H. pylori* antibodies in relation to analyzed independent factors.

Characteristics	HP+		HP−		*p*-Value
	*n*	%	*n*	%	
**Education**					
Basic	216	73.2 ^a^	79	26.8	<0.001
Intermediate	49	57.6	36	42.4	
Advanced	325	61.1	207	38.9	
**Childhood living environment**					
Urban	442	63.5	254	36.5	0.178
Rural	148	68.5	68	31.5	
**Drinking water sourced from a municipal supply**					
Yes	419	63.3	254	37.7	0.010
No	171	71.5	68	28.5	
**Childhood access to hot water**					
Yes	418	62.4	252	37.6	0.015
No	172	71.1	70	28.9	
**Number of household members in childhood**					
2–3	112	60.9	72	39.1	0.353
4	283	64.6	155	35.4	
5 and more	196	67.4	95	32.6	

HP+: antibodies against *H. pylori* detected; HP−: antibodies against *H. pylori* not detected. ^a^ *p* < 0.05, compared to intermediate and advanced education in the HP+ group (Z-test with Bonferroni correction).

**Table 4:** Odds ratios of *H. pylori* infection in relation to analyzed factors (univariable and multivariable logistic regression analyses).

Factors	Univariable Analysis			Multivariable Analysis		
	OR	95% CI	*p*-Value	OR	95% CI	*p*-Value
**Sex**						
Females	1			1		
Males	1.34	1.02–1.76	0.034	1.32	1.0–1.75	0.049
Age *	1.03	1.02–1.04	<0.001	1.03	1.01–1.04	<0.001
**Education**						
Basic	1			1		
Intermediate	0.50	0.30–0.82	0.006	0.45	0.27–0.75	0.002
Advanced	0.57	0.42–0.78	<0.001	0.65	0.47–0.89	0.008
**Childhood living environment**						
Urban	1			1		
Rural	1.25	0.90–1.73	0.179	1.03	0.72–1.48	0.876
**Drinking water sourced from a municipal supply**						
Yes	1			1		
No	1.48	1.08–2.04	0.016	1.20	0.64–1.57	0.399
**Childhood access to hot water**						
Yes	1			1		
No	1.52	1.11–2.10	0.010	0.98	0.64–1.51	0.931

* Change in odds ratio per one-year increase in age; 95% CI: 95% confidence interval.

**Table 5:** Distribution of study subjects based on previous *H. pylori* serologic testing.

History of *H. pylori* Serologic Testing	Males		Females		Total		*p*-Value Between Males and Females
	*n*	%	*n*	%	*n*	%	
Not tested	395	75.2	342	65.8	737	70.5	<0.001
Tested—negative	47	9.0	74	14.2	121	11.6	
Tested—positive	61	11.6	96	18.5	157	15.0	
Unknown	22	4.2	8	1.5	30	2.9	
**Total**	**525**	**100**	**520**	**100**	**1046**	**100**	

## Data Availability

The datasets used and/or analyzed during the current study are available from the corresponding author upon reasonable request.

## Abbreviations

The following abbreviations are used in this manuscript:

*H. pylori*	*Helicobacter pylori*
GC	Gastric cancer
PUD	Peptic ulcer disease
IgG	Immunoglobulin G
IARC	International Agency for Research on Cancer
GLOBOCAN	Global Cancer Observatory
95% CI	95% confidence interval
OR	Odds ratio

## References

[1] Malfertheiner P., Megraud F., Rokkas T., Gisbert J.P., Liou J.-M., Schulz C., Gasbarrini A., Hunt R.H., Leja M., O’Morain C. (2022). Management of *Helicobacter pylori* infection: The Maastricht VI/Florence consensus report. Gut.

[2] Chey W.D., Howden C.W., Moss S.F., Morgan D.R., Greer K.B., Grover S., Shah S.C. (2024). ACG Clinical Guideline: Treatment of *Helicobacter pylori* Infection. Am. J. Gastroenterol..

[3] Malfertheiner P., Camargo M.C., El-Omar E., Liou J.-M., Peek R., Schulz C., Smith S.I., Suerbaum S. (2023). *Helicobacter pylori* infection. Nat. Rev. Dis. Prim..

[4] Ahn H.J., Lee D.S. (2015). *Helicobacter pylori* in gastric carcinogenesis. World J. Gastrointest. Oncol..

[5] Bray F., Laversanne M., Sung H., Ferlay J., Siegel R.L., Soerjomataram I., Jemal A. (2024). Global cancer statistics 2022: GLOBOCAN estimates of incidence and mortality worldwide for 36 cancers in 185 countries. CA Cancer J. Clin..

[6] Hooi J.K.Y., Lai W.Y., Ng W.K., Suen M.M.Y., Underwood F.E., Tanyingoh D., Malfertheiner P., Graham D.Y., Wong V.W.S., Wu J.C.Y. (2017). Global Prevalence of *Helicobacter pylori* Infection: Systematic Review and Meta-Analysis. Gastroenterology.

[7] Chen Y.-C., Malfertheiner P., Yu H.-T., Kuo C.-L., Chang Y.-Y., Meng F.-T., Wu Y.-X., Hsiao J.-L., Chen M.-J., Lin K.-P. (2024). Global Prevalence of *Helicobacter pylori* Infection and Incidence of Gastric Cancer Between 1980 and 2022. Gastroenterology.

[8] Brown L.M. (2000). *Helicobacter pylori*: Epidemiology and routes of transmission. Epidemiol. Rev..

[9] Leja M., Cine E., Rudzite D., Vilkoite I., Huttunen T., Daugule I., Rumba-Rozenfelde I., Pimanov S., Liepniece-Karele I., Pahomova J. (2012). Prevalence of *Helicobacter pylori* infection and atrophic gastritis in Latvia. Eur. J. Gastroenterol. Hepatol..

[10] Thjodleifsson B., Asbjörnsdottir H., Sigurjonsdottir R.B., Gíslason D., Olafsson I., Cook E., Jogi R., Janson C. (2007). Seroprevalence of *Helicobacter pylori* and cagA antibodies in Iceland, Estonia and Sweden. Scand. J. Infect. Dis..

[11] Celiński K., Kurzeja-Mirosław A., Słomka M., Cichoz-Lach H., Madro A., Kasztelan-Szczerbińska B. (2006). The effects of environmental factors on the prevalence of *Helicobacter pylori* infection in inhabitants of Lublin Province. Ann. Agric. Environ. Med..

[12] Ruibys G., Denapiene G., Wright R.A., Irnius A. (2004). Prevalence of *Helicobacter pylori* Infection in Lithuanian Children. Am. J. Gastroenterol..

[13] Kupcinskas J., Leja M. (2014). Management of *Helicobacter pylori*-Related Diseases in the Baltic States. Dig. Dis..

[14] Jonaitis L., Ivanauskas A., Janciauskas D., Funka K., Sudraba A., Tolmanis I., Krams A., Stirna D., Vanags A., Kupcinskas L. (2007). Precancerous gastric conditions in high *Helicobacter pylori* prevalence areas: Comparison between Eastern European (Lithuanian, Latvian) and Asian (Taiwanese) patients. Medicina.

[15] Jonaityte I.R., Ciupkeviciene E., Jonaitis P., Kupcinskas J., Petkeviciene J., Jonaitis L. (2021). Changes in the Seroprevalence of *Helicobacter pylori* among the Lithuanian Medical Students over the Last 25 Years and Its Relation to Dyspeptic Symptoms. Medicina.

[16] Leja M. (2024). Where are we with gastric cancer screening in Europe in 2024?. Gut.

[17] Jonaitis P., Kupčinskas J., Nyssen O.P., Puig I., Gisbert J.P., Jonaitis L.V. (2021). Evaluation of the Effectiveness of *Helicobacter Pylori* Eradication Regimens in Lithuania During the Years 2013–2020: Data from the European Registry on *Helicobacter pylori* Management (Hp-EuReg). Medicina.

[18] Laszewicz W., Iwańczak F., Iwańczak B. (2014). Seroprevalence of *Helicobacter pylori* infection in Polish children and adults depending on socioeconomic status and living conditions. Adv. Med. Sci..

[19] Kondratiuk N., Paliy I., Zaika S. (2021). Analysis of the prevalence of *Helicobacter pylori* infection and the effectiveness of eradication schemes in patients with the upper gastrointestinal tract disorders (according to the results of 13C-urea breath tests for 2006–2019). Prz. Gastroenterol..

[20] Razuka-Ebela D., Polaka I., Parshutin S., Santare D., Ebela I., Murillo R., Tzivian L., Park J.Y., Leja M. (2020). Sociodemographic, Lifestyle and Medical Factors Associated with *Helicobacter pylori* Infection. J. Gastrointest. Liver Dis..

[21] Taylor C.S., McMahon M.V., Ward Z.J., Alarid-Escudero F., Camargo M.C., Laszkowska M., Roa J., Yeh J.M. (2025). Birth cohort and age-specific trends in global *Helicobacter pylori* seroprevalence: A scoping review. Lancet Reg. Health-Am..

[22] Wernly S., Semmler G., Rezar R., Schaffler-Schaden D., Flamm M., Aigner E., Datz C., Wernly B. (2024). Assessing the association between *H. pylori* infection and educational status: Implications for screening strategies?. Minerva Gastroenterol..

[23] Park J.S., Jun J.S., Seo J.-H., Youn H.-S., Rhee K.-H. (2021). Changing prevalence of *Helicobacter pylori* infection in children and adolescents. Clin. Exp. Pediatr..

[24] Li Y., Choi H., Leung K., Jiang F., Graham D.Y., Leung W.K. (2023). Global prevalence of *Helicobacter pylori* infection between 1980 and 2022: A systematic review and meta-analysis. Lancet Gastroenterol. Hepatol..

[25] Borka Balas R., Meliț L.E., Mărginean C.O. (2022). Worldwide Prevalence and Risk Factors of *Helicobacter pylori* Infection in Children. Children.

[26] Amaral O., Fernandes I., Veiga N., Pereira C., Chaves C., Nelas P., Silva D. (2017). Living Conditions and *Helicobacter pylori* in Adults. Biomed Res. Int..

[27] Contreras M., Fernández-Delgado M., Reyes N., García-Amado M.A., Rojas H., Michelangeli F. (2015). *Helicobacter pylori* Infection in Rural and Urban Dyspeptic Patients from Venezuela. Am. J. Trop. Med. Hyg..

[28] Rosenstock S., Kay L., Rosenstock C., Andersen L.P., Bonnevie O., Jørgensen T. (1997). Relation between *Helicobacter pylori* infection and gastrointestinal symptoms and syndromes. Gut.

[29] Marzio L., Cappello G., Ballone E. (2003). Evaluation of dyspeptic symptoms in patients with and without *Helicobacter pylori* infection and normal upper gastrointestinal endoscopy. Dig. Liver Dis..

[30] O’Connor A., O’Morain C.A., Ford A.C. (2017). Population screening and treatment of *Helicobacter pylori* infection. Nat. Rev. Gastroenterol. Hepatol..

[31] Januszewicz W., Turkot M.H., Malfertheiner P., Regula J. (2023). A Global Perspective on Gastric Cancer Screening: Which Concepts Are Feasible, and When?. Cancers.

[32] Chiang T.-H., Cheng H.-C., Chuang S.-L., Chen Y.-R., Hsu Y.-H., Hsu T.-H., Lin L.-J., Lin Y.-W., Chu C.-H., Wu M.-S. (2022). Mass screening and eradication of *Helicobacter pylori* as the policy recommendations for gastric cancer prevention. J. Formos. Med. Assoc..

